# Signs of Deregulated Gene Expression Are Present in Both CD14^+^ and CD14^-^ PBMC From Non-Obese Men With Family History of T2DM

**DOI:** 10.3389/fendo.2020.582732

**Published:** 2021-02-15

**Authors:** Michal Koc, Michaela Šiklová, Veronika Šrámková, Marek Štěpán, Eva Krauzová, Vladimír Štich, Lenka Rossmeislová

**Affiliations:** ^1^ Department for Pathophysiology, Centre for Research on Nutrition, Metabolism and Diabetes, Third Faculty of Medicine, Charles University, Prague, Czechia; ^2^ Franco-Czech Laboratory for Clinical Research on Obesity, Third Faculty of Medicine, Prague, and Inserm, Toulouse, France; ^3^ Department of Internal Medicine, Third Faculty of Medicine, Charles University, and Kralovske Vinohrady University Hospital, Prague, Czechia

**Keywords:** oral glucose tolerance test, first-degree relatives, gene expression, peripheral blood mononuclear cells, type 2 diabetes mellitus, CD14^−^ cells, CD14^+^ cells

## Abstract

**Aim:**

Development of type 2 diabetes (T2DM) is associated with disturbances in immune and metabolic status that may be reflected by an altered gene expression profile of peripheral blood mononuclear cells (PBMC). To reveal a potential family predisposition to these alterations, we investigated the regulation of gene expression profiles in circulating CD14^+^ and CD14^-^ PBMC in fasting conditions and in response to oral glucose tolerance test (OGTT) in glucose tolerant first-degree relatives (FDR) of T2DM patients and in control subjects.

**Materials and Methods:**

This work is based on the clinical study LIMEX (NCT03155412). Non-obese 12 non-diabetic (FDR), and 12 control men without family history of diabetes matched for age and BMI underwent OGTT. Blood samples taken before and at the end of OGTT were used for isolation of circulating CD14^+^ and CD14^-^ PBMC. In these cells, mRNA levels of 94 genes related to lipid and carbohydrate metabolism, immunity, and inflammation were assessed by qPCR.

**Results:**

Irrespectively of the group, the majority of analyzed genes had different mRNA expression in CD14^+^ PBMC compared to CD14^-^ PBMC in the basal (fasting) condition. Seven genes (IRS1, TLR2, TNFα in CD14^+^ PBMC; ABCA1, ACOX1, ATGL, IL6 in CD14^-^ PBMC) had different expression in control vs. FDR groups. OGTT regulated mRNA levels of nine genes selectively in CD14^+^ PBMC and of two genes (ABCA1, PFKL) selectively in CD14^-^PBMC. Differences in OGTT-induced response between FDR and controls were observed for EGR2, CCL2 in CD14^+^ PBMC and for ABCA1, ACOX1, DGAT2, MLCYD, and PTGS2 in CD14^-^ PBMC.

**Conclusion:**

This study revealed a different impact of glucose challenge on gene expression in CD14^+^ when compared with CD14^-^ PBMC fractions and suggested possible impact of family predisposition to T2DM on basal and OGTT-induced gene expression in these PBMC fractions. Future studies on these putative alterations of inflammation and lipid metabolism in fractionated PBMC in larger groups of subjects are warranted.

## Introduction

Type 2 diabetes mellitus (T2DM) is associated with a number of disturbances of immune status and metabolic pathways (1) that can be detected not only in insulin sensitive tissue but also in easily accessible peripheral blood mononuclear cells (PBMC). PBMC from patients with T2DM have shown alterations in mRNA levels of genes involved in the regulation of inflammation, lipid and glucose metabolism, and several signaling pathways (2–5). Development of T2DM is based on both environmental and genetic factors. While the obesogenic environment and the subsequent development of obesity is one of the most important factors contributing to the development of T2DM (6), the heritability of T2DM reaches 20%–80%, when first-degree relatives (FDR) of T2DM patients are about 3 times more likely to develop the disease than individuals without a positive family history of the disease (7). Thus, it might be hypothesized that alterations in gene expression are present already in non-obese individuals with genetic predisposition to T2DM, such as FDR. Indeed, a number of genes with dysregulated expression was found in FDR when compared with subjects without T2DM in antecedence, including adiponectin in adipose tissue (8) and genes involved in insulin signaling and fatty acid metabolism in skeletal muscle (9, 10). Although PBMC proved to be a good surrogate marker of systemic and adipose tissue metabolic state (11, 12), they represent a mixture of cell types with considerably different roles, behavior and metabolism, i.e. monocytes, dendritic cells and lymphocytes (13) and analysis of PBMC population in whole may limit the interpretation of results.

Therefore, the aim of our study was to examine the gene expression profile in two major fractions of PBMC: CD14 positive (i.e. mostly monocytes, representing 10%–20% of PBMC) and CD14 negative (i.e. mostly lymphocytes and natural killer cells, representing 70%–90% and 5%–10% of PBMC, resp.) in FDR in comparison with healthy controls without diabetic relatives so that the differential behavior of innate and acquired immunity systems could be revealed. The selected genes covered immunity and inflammation pathways and pathways of lipid and carbohydrate metabolism. Since we hypothesized that the nutrient-induced response of the genes might have a higher discriminative power than baseline values when comparing FDR to the control group, we analyzed the possible alteration of mRNA expression in these two PBMC fractions also in response to the nutritional challenge represented by OGTT.

## Material and Methods

### Subjects Characteristics

This work is based on the clinical study LIMEX (NCT03155412) including 51 healthy non-obese men. The two groups of men - 1) non-diabetic first-degree relatives of T2DM patients (FDR); 2) control group - subjects without any family history of diabetes (CON) - were matched for age and BMI. Family history of diabetes was considered as follows: two first-degree relatives (parents, siblings) or one first-degree and one or more second-degree relatives (grandparents, uncle, aunt) that were diagnosed with T2DM. Exclusion criteria for both groups were: body weight change more than 3 kg within 3 months preceding the study, smoking, any medication, hypertension, hyperlipidaemia, and drug or alcohol abuse.

Subjects were examined at 8.00 h in the fasting state. Body weight, waist, and hip circumferences were measured. Body composition was assessed by bioimpedance (QuadScan 4000, Bodystat, Douglas, British Isles). All men underwent an initial examination consisting of OGTT. Based on OGTT results eight subjects with impaired glucose tolerance were excluded. PBMC were isolated from a subgroup of 37 men (control, n=18; FDR, n=19). 13 subjects (control, n =6; FDR, n=7) were excluded because of the insufficient amount of isolated RNA from at least one fraction of PBMC in either of the time points of OGTT. Gene expression analysis in PBMC was performed in 12 subjects from the control group and 12 subjects from FDR group.

### Oral Glucose Tolerance Test

Seventy-five grams of glucose was administered orally and blood samples for routine analysis were taken at the time points 0, 30, 60, 90, and 120 min. PBMC were isolated from 9 ml of full blood in time points 0 and 120 min and immunoseparated into CD14 positive and negative subpopulations using Dynabeads CD14 (Thermo Fisher, MA USA) as described before (14).

### Plasma Analysis

Plasma samples were prepared from uncoagulated peripheral blood by centrifugation. Plasma glucose was determined with a glucose oxidase technique (Beckman Instruments, Fullerton, CA). Plasma insulin was measured using an Insulin Irma kit (Immunotech, Prague, Czech Republic). Lipid concentrations were determined using standard biochemical methods in certified laboratories.

### Gene Expression Analysis

Total RNA was isolated from CD14^+^ and CD14^-^ subpopulation using an RNeasy Mini kit (Qiagen, Germany). RNA concentration was measured using Nanodrop1000 (Thermo Fisher Scientific, USA). To remove genomic DNA, DNAse I (Invitrogen, USA) treatment was applied. 300 ng of total RNA was reversely transcribed using High Capacity cDNA Reverse Transcription Kit (Applied Biosystems, USA). For microfluidics, 4 ng of cDNA were preamplified within 18 cycles to improve the detection of target genes during subsequent Real Time qPCR (TaqMan Pre Amp Master Mix Kit, Applied Biosystems, USA). For the preamplification, TaqMan gene expression assays of all target genes (**Supplemental Table 1**) were pooled together and diluted with water to the final concentration 0.2× for each probe. Samples with preamplified cDNAs were diluted 20 times, qPCR was then performed on Biomark Real Time qPCR system using a 96 × 96 array (Fluidigm, USA). Expression of seven genes (AKT1, APOE, CXCL1, GZMB, MMP3, SLC2A4, MLXIPL) was either below the detection limit or their amplification curves did not pass the initial quality test (**Supplementary Table 2**). Data were normalized to geomean of two reference genes (RPS13, TBP), 2^-ΔCt^ was calculated and used for statistical analysis. Genes with median of 2^-ΔCt^ below 0.0001 were considered as genes with insignificant level of expression and excluded from the analysis (CLIC3, IL2, IL6, KIR2DL4, MMP2, SLC27A2, VCAM1 in CD14^+^ PBMC; CCL2, FOS, IL10, IL2, MMP2, PPARγ, SLC27A2, VCAM1 in CD14^-^ PBMC). Outliers were identified by ROUT method, with Q value set to 0.5%. Genes with more than three missing values per group were excluded from analysis (ACOX1, COX6C, and DGAT1 in CD14^+^ PBMC). The effect of OGTT was in figures expressed as the median of fold change – OGTT vs. basal- of each subject in the control and FDR group.

### Statistical Analysis

Data are presented as means ± SEM (anthropometric and biochemical variables) or median with 25^th^ and 75^th^ percentile. Statistical analysis was performed using GraphPad Prism 9.0 for Windows (LaJolla, USA). Differences in the baseline values of the measured anthropometric, and biochemical variables were evaluated by Multiple Unpaired t test with multiple comparisons by False Discovery Rate (method by Benjamini, Krieger, and Yekutieli) set to 5%. Differences in mRNA levels (2^-ΔCt^) and responses to OGTT (mRNA fold changes) between groups (FDR vs. controls) were evaluated by Multiple Mann-Whitney tests, with multiple comparisons by False Discovery Rate (method by Benjamini, Krieger, and Yekutieli) set to 5%. Evolution of log2 transformed glucose and insulin levels during OGTT was evaluated by Two Way ANOVA with Sidak multiple comparison test. Effects of OGTT on mRNA levels within each of the two groups were evaluated by Multiple Wilcoxon matched pairs signed rank tests, with multiple comparisons by False Discovery Rate (method by Benjamini, Krieger, and Yekutieli) set to 5%. The Spearman rank-order correlation coefficient was calculated in correlation analysis. The level of significance was set at p <0.05 and q value <0.05.

## Results

### Subjects Characteristics

Anthropometric and laboratory characteristics of both groups of subjects are presented in **Table 1**. The groups were not different in respect to age, body weight, waist circumference, BMI, relative fat mass, fat free mass, plasma triacylglycerol (TAG), LDL, cholesterol and uric acid. Plasma HDL was higher in the control group. Fasting plasma glucose and insulin were higher in FDR. Similarly, plasma glucose and insulin were higher at 120 min of OGTT. Time evolution of glucose and insulin levels during OGTT are shown in **Supplemental Figure 1**. Calculated indices of insulin resistance, HOMA-IR and Matsuda index (15) suggested lower whole body insulin sensitivity in FDR.

**Table 1:** Anthropometric and biochemical characteristics of the subjects.

	Controls (n = 12)	FDR (n=12)	p value	q value
Age (years)	34.3± 3.5	37.0 ± 3.8	NS	NS
Body weight (kg)	82.6 ± 2.1	86.2 ± 1.2	NS	NS
BMI (kg/m^2^)	24.7 ± 0.5	26.0 ± 0.5	NS	NS
Waist circumference (cm)	84.0 ± 1.2	88.0 ± 1.6	NS	NS
Fat Mass (%)	17.8 ± 3.4	17.8 ± 5.4	NS	NS
Fat Mass (kg)	13.6 ± 1.0	16.9 ± 1.0	NS	NS
Fat Free Mass (kg)	68.8 ± 1.2	68.6 ± 1.0	NS	NS
Cholesterol (mmol/l)	4.34 ± 0.2	5.05 ± 0.27	NS	NS
HDL cholesterol (mmol/l)	1.43 ± 0.05	1.09 ± 0.06	0.005	0.021
LDL cholesterol (mmol/l)	2.41 ± 0.16	3.05 ± 0.2	NS	NS
TAG (mmol/l)	1.17 ± 0.27	1.89 ± 0.43	NS	NS
Uric Acid (µmol/l)	342 ± 19	336 ± 13	NS	NS
Fasting glucose (mmol/l)	5.10 ± 0.07	5.61 ± 0.10	0.006	0.021
Fasting insulin (mU/l)	5.30 ± 0.67	10.93 ± 1.10	0.002	0.016
Glucose at 120 min of OGTT (mmol/l)	4.80 ± 0.20	6.17 ± 0.26	0.006	0.021
Insulin at 120 min of OGTT (mU/l)	17.20 ± 5.90	43.01 ± 6.94	0.021	NS
Glucose AUC OGTT (mmol-h/l)	770 ± 24	860 ± 29	NS	NS
Insulin AUC OGTT(mU-h/l)	4381 ± 333	7212 ± 883	0.05	NS
HOMA-IR	1.21 ± 0.16	2.79 ± 0.32	0.002	0.016
Matsuda index	9.91 ± 1.83	5.71 ± 0.97	0.023	NS

Data are shown as mean ± SEM. Statistical difference between the groups evaluated by Multiple Unpaired t tests, with multiple comparisons by Benjamini, Krieger, and Yekutieli method of False Discovery Rate. Level of significance for both p and q value was set to <0.05. AUC, area under the curve; BMI, Body mass index; FDR, first degree relatives of T2DM patients; HDL, high density lipoprotein; HOMA-IR, homeostasis model assessment of insulin resistance; LDL, low density lipoproteins; NS, not significant; TAG, triglycerides.

### Basal Expression in PBMC Subpopulations

*Comparison CD14^+^ vs. CD14^-^ PBMC.* When gene expression was analyzed in all samples, irrespectively of the group, the majority of genes were differentially expressed in CD14^+^ vs. CD14^-^ PBMC (only four genes had a similar expression level in both PBMC fractions) (**Supplemental Table 1**). Four genes (CCL2, FOS, IL10, and PPARγ) were detected only in CD14^+^ PBMC and six genes (CD36, EGR1, EGR2, IL1β, TLR2, TLR4) had more than 20 times higher level of expression in CD14^+^ vs. CD14^-^ PBMC. Six genes (ACOX1, CLIC3, COX6C, DGAT1, IL6, KIR2DL4) were detected only in CD14^-^ PBMC and three genes (CCL5, PRF1, and SLC2A1) had more than 20 times higher level of expression in CD14^-^ vs. CD14^+^ PBMC (**Supplemental Table 1**).

**Figure 1:**
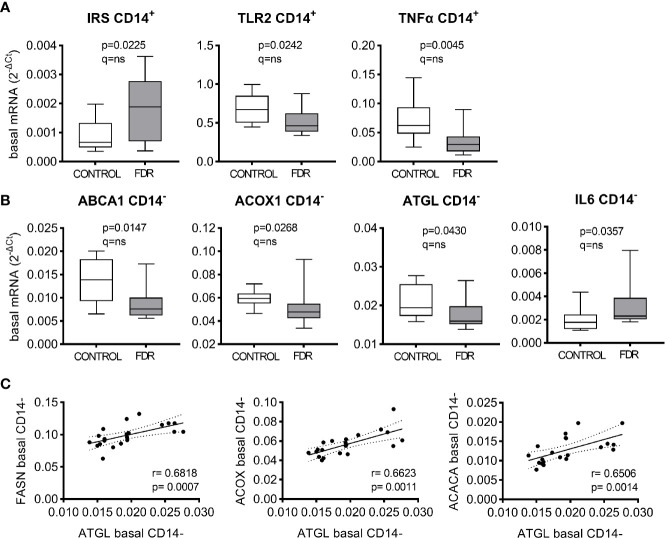
Effect of group (FDR vs. controls) on gene expression in CD14^+^ **(A)** and CD14^-^ **(B)** peripheral blood mononuclear cells (PBMC) in the fasting (basal) conditions. Gene expression was normalized to the geomean of reference genes RPS13 and TBP and evaluated by Multiple Mann-Whitney tests, with multiple comparisons by False Discovery Rate (method by Benjamini, Krieger, and Yekutieli). Level of significance for both p and q value was set to <0.05. Box plots show the medians with 25^th^ and 75^th^ percentiles, whiskers show min and max values. **(C)** Correlations of basal gene expressions (2^-ΔCt^) of ATGL and other genes involved in lipid handling in CD14^-^ PBMC. The Spearman rank-order correlation coefficient is shown.

*Comparison FDR vs. Controls.* In the basal (fasting) state, majority of the analyzed genes in PBMC subpopulations had similar mRNA levels in control and FDR groups, except for seven genes. CD14^+^ PBMC exhibited lower basal mRNA expression of TLR2 and TNFα genes and higher expression of IRS1 gene in FDR group (**Figure 1A**). Expression of neither of these genes correlated with HOMA-IR or Matsuda index (not shown). CD14^-^ PBMC isolated from FDR exhibited lower basal expression of ABCA1, ACOX1, and ATGL and higher expression of IL6 when compared to controls (**Figure 1B**). Expression of ATGL (in CD14^-^ PBMC from all subjects) correlated with the expression of markers of *de novo* lipogenesis (FASN, ACACA) and β oxidation (ACOX1) (**Figure 1C**). However, when applying correction for multiple testing we did not confirm these seven genes as significantly different between the groups.

### Effect of OGTT on Gene Expression in PBMC Subpopulations

mRNA expression of pyruvate dehydrogenase kinase isozyme 4 (PDK4) was used as a positive control for the OGTT response since glucose inhibits PDK4 expression (12, 16). A significant down-regulation of PDK4 mRNA expression in response to OGTT was found in both experimental groups as well as in both PBMC fractions.

**Figure 2:**
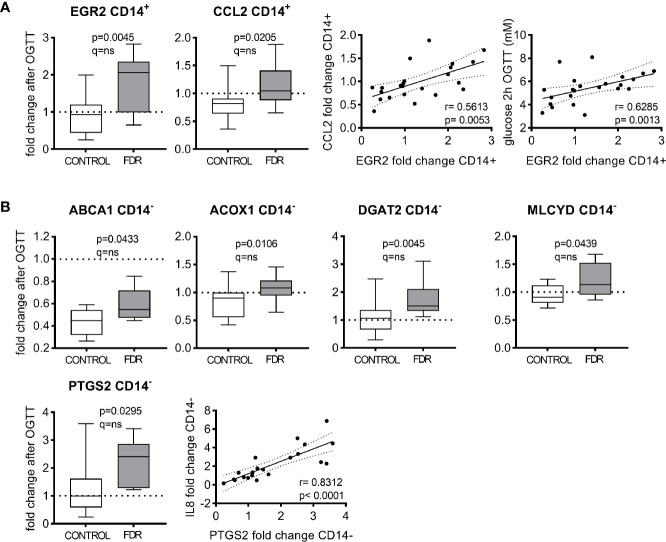
Effect of group (FDR vs controls) on oral glucose tolerance test (OGTT) –induced gene expression changes in circulating CD14^+^ **(A)** and CD14^-^ PBMC **(B)**. The effect is presented as fold change (OGTT vs. basal) in each group. Gene expression was normalized to the geomean of reference genes RPS13 and TBP. Box plots show the medians with 25^th^ and 75^th^ percentiles, whiskers show min and max values. Effects of OGTT on mRNA levels within individual groups were evaluated by Multiple Mann-Whitney tests, with multiple comparisons by False Discovery Rate (method by Benjamini, Krieger, and Yekutieli). Correlations of changes in gene expressions (fold change) and glucose levels at the end of OGTT (Spearman rank-order correlation coefficient) are shown. Level of significance for both p and q value was set to <0.05.

In CD14^+^ PBMC, 21 genes were regulated in response to OGTT in at least one group of subjects (**Table 2**). Among them, 11 genes were significantly changed in both groups. In nine genes (CD36, DUSP1, GOT2, HIF1α, ICAM1, IRS1, PCK2, TCF7L2, and TGFβ1) the response to OGTT was specific for CD14^+^ fraction (i.e. the change was not present in CD14^-^ PBMC). Only EGR2 and CCL2 genes were differentially regulated in FDR vs. controls in response to OGTT, i.e. fold changes of mRNA were higher in FDR compared to controls (**Figure 2A**). In the case of EGR2, mRNA expression was significantly upregulated in FDR but not control group (**Table 2**). OGTT induced changes of mRNA levels of these two genes were positively correlated with each other and the change of EGR2 was correlated with 2h OGTT glucose levels (**Figure 2A**). However, it should be noted that the significant differences in the OGTT-induced response between the groups (FDR vs. control) were not confirmed after correction for multiple testing.

**Table 2:** Effect of oral glucose tolerance test (OGTT) on gene expression.

CD14^+^ PBMC	Control				FDR				Group difference	
Gene	Fold change	75^th^ - 25^th^ percentiles	p value	q value	Fold change	75^th^ - 25^th^ percentiles	p value	q value	p value	q value
ACACA	0.873	0.993-0.691	0.0269	NS	1.497	2.997-0.541	0.0015	0.0104	NS	NS
**CCL2**	0.820	0.908-0.639	NS	NS	1.046	1.412-0.877	NS	NS	0.0205	NS
CD36	1.344	1.595-1.076	0.0034	0.0230	1.226	1.423-1.072	0.001	0.0078	NS	NS
CPT1α	0.682	1.131-0.548	0.0210	NS	0.696	0.906-0.600	0.0034	0.0182	NS	NS
DUSP1	1.148	1.941-1.079	0.0093	0.0445	1.296	1.432-1.049	0.0034	0.0182	NS	NS
**EGR2**	0.931	1.203-0.444	NS	NS	2.060	2.352-0.991	0.0137	0.0486	0.0045	NS
GOT2	1.078	1.132-1.028	0.0034	0.0230	1.130	1.269-1.073	0.001	0.0078	NS	NS
HIF1α	0.905	0.928-0.732	0.0068	0.0353	0.785	0.853-0.652	0.0137	0.0486	NS	NS
ICAM1	1.215	1.437-1.083	0.0020	0.0230	1.156	1.329-1.084	0.0093	0.0371	NS	NS
IL10	1.874	2.543-1.065	0.0420	NS	2.693	3.668-1.132	0.0068	0.0313	NS	NS
IRS1	1.462	1.715-1.387	0.0024	0.0230	1.367	1.598-1.277	0.001	0.0078	NS	NS
JUN	1.237	1.547-1.187	0.0068	0.0353	1.188	1.556-0.968	NS	NS	NS	NS
MMP9	1.285	2.046-0.892	NS	NS	1.509	2.154-1.342	0.001	0.0078	NS	NS
p53	1.116	1.149-1.043	0.0029	0.0230	1.039	1.143-0.935	NS	NS	NS	NS
PCK2	1.244	1.299-1.161	0.0005	0.0164	1.193	1.331-1.129	0.0005	0.0078	NS	NS
PDK4	0.201	0.264-0.079	0.005	0.0164	0.143	0.250-0.088	0.0005	0.0078	NS	NS
PLIN2	0.809	0.887-0.617	0.001	0.0219	0.700	0.818-0.595	0.0005	0.0078	NS	NS
PPARα	1.088	1.158-1.028	0.0244	NS	1.169	1.243-1.083	0.0034	0.0182	NS	NS
SLC27A1	1.147	1.288-1.088	0.0068	0.0353	1.065	1.262-.966	NS	NS	NS	NS
SLC2A1	1.217	1.333-0.995	0.0210	NS	1.281	1.723-1.050	0.0068	0.0313	NS	NS
TCF7L2	1.453	1.631-1.207	0.0015	0.0230	1.511	1.585-1.224	0.001	0.0078	NS	NS
TGF-β1	1.108	1.164-1.040	0.0034	0.0230	1.069	1.164-1.000	0.0093	0.0371	NS	NS
CD14^+^ PBMC	Control				FDR				Group difference	
Gene	Fold change	75^th^ - 25^th^ percentiles	p value	q value	Fold change	75^th^ - 25^th^ percentiles	p value	q value	p value	q value
**ABCA1**	0.448	0.544-0.320	0.002	0.0167	0.546	0.718-0.474	0.002	0.0379	0.0433	NS
**ACOX1**	0.899	1.002-0.558	0.0342	NS	1.080	1.217-0.946	NS	NS	0.0106	NS
ATF4	1.125	1.200-1.034	0.0024	0.0167	1.231	1.402-1.031	0.0098	NS	NS	NS
CPT1α	0.739	0.885-0.485	0.0024	0.0167	0.723	0.985-0.653	0.0322	NS	NS	NS
**DGAT2**	1.070	1.356-0.660	NS	NS	1.511	2.118-1.323	0.001	0.0379	0.0045	NS
EGR2	1.723	2.231-1.212	0.0015	0.0167	1.338	2.193-0.924	NS	NS	NS	NS
H6PD	1.261	1.572-1.072	0.0029	0.0182	1.285	1.350-0.941	NS	NS	NS	NS
IL8	1.070	3.691-0.629	NS	NS	2.927	4.349-1.767	0.0029	0.0455	NS	NS
JUN	1.230	1.387-1.136	0.0049	0.0256	1.137	1.385-0.856	NS	NS	NS	NS
KCNQ1	1.127	1.337-0.993	0.0068	0.03333	1.259	1.354-1.017	NS	NS	NS	NS
KIR2DL4	0.534	0.790-0.321	0.0039	0.0222	0.686	1.412-0.494	NS	NS	NS	NS
**MLCYD**	0.910	1.123-0.808	NS	NS	1.136	1.527-0.952	NS	NS	0.0439	NS
PDK4	0.285	0.488-0.187	0.0005	0.0111	0.245	0.412-0.170	0.001	0.0379	NS	NS
PFKL	0.765	0.794-0.695	0.001	0.0133	0.674	0.791-0.579	0.0039	NS	NS	NS
PLIN2	0.793	0.897-0.724	0.001	0.0133	0.758	0.971-0.711	0.0068	NS	NS	NS
**PTGS2**	0.989	1.618-0.583	NS	NS	2.402	2.862-1.273	0.002	0.0379	0.0295	NS
SREBP1	1.313	1.486-1.153	0.0005	0.0111	1.219	1.390-1.106	0.0322	NS	NS	NS
STAT1	1.170	1.250-1.127	0.0005	0.0111	1.216	1.318-1.144	NS	NS	NS	NS
TNFα	1.922	3.145-1.136	0.0024	0.0167	1.614	2.227-0.615	NS	NS	NS	NS

OGTT – induced regulation of gene expression is expressed as a fold change in circulating CD14^+^ PBMC and CD14^-^ PBMC in first degree relatives of T2DM diabetic patients (FDR) and in controls. Data are presented as median and 75^th^–25^th^ percentile range of fold change (2h OGTT vs. basal gene expression). Effects of OGTT on mRNA levels within individual groups were evaluated by paired Multiple Wilcoxon matched pairs signed rank tests, Multiple Mann-Whitney tests were used to analyze difference in OGTT-induced fold change between the groups. Both tests were corrected to multiple comparisons by False Discovery Rate (method by Benjamini, Krieger, and Yekutieli). Level of significance for both p and q value was set to <0.05.

FDR, first-degree relatives of T2DM patients; NS, not significant; PBMC, peripheral blood mononuclear cells. Genes found to have a differential response to OGTT in FDR vs. controls are in bold.

In CD14^-^ PBMC, 17 genes were regulated in response to OGTT in at least one group of subjects (**Table 2**), when two genes (ABCA1, PDK4) were significantly changed in both groups. This change was specific for CD14^-^ PBMC fraction in the case of ABCA1. There were five genes for which the OGTT-induced change in expression was different in the FDR group when compared with controls (**Figure 2B**). Out of these genes, fold changes of ACOX1, DGAT2, MLCYD, and PTGS2 mRNA were higher in FDR compared to controls. In the case of DGAT2 and PTGS2 mRNA expression was significantly upregulated in FDR but not control group (**Table 2**). ABCA1 was down-regulated in both groups, the downregulation being less pronounced in FDR (**Figure 2B**, **Table 2**). OGTT induced changes of PTGS2 mRNA were positively correlated with the changes of another pro-inflammatory gene IL8 (**Figure 2B**). However the correction for multiple testing did not confirm that the change of mRNA expression of above described genes in response to OGTT was significantly different between FDR and control subjects.

## Discussion

In this study we investigated the impact of family history of T2DM on mRNA gene expression in circulating CD14^+^ and CD14^-^ PBMC in the fasting conditions and in response to OGTT. FDR subjects included in our study were non-obese and with normal glucose tolerance, but they already showed markers of metabolic impairment: they had higher fasting and post-OGTT glucose and insulin levels and higher HOMA-IR index. Thus, blood cells of these FDR are chronically exposed to mildly higher insulin and glucose levels and, consequently, may react differently to glucose challenge. Indeed, our study suggested that several genes could have different basal expression and/or could be differentially regulated in response to OGTT in FDR when compared to controls, in agreement with several previous studies showing that the changes of gene expression in PBMC induced by glucose intake reflect the metabolic status of the individual (12, 16, 17).

Importantly, we demonstrated that the detected alterations in basal gene expression and its OGTT-induced regulation were different in respect to the type of immune cell population, i.e. CD14^+^ and CD14^-^ PBMC. Among the genes that could be considered as markers of one of the PBMC fractions (i.e., they were expressed only in one fraction or had more than 20 times higher/lower level of expression in CD14^+^ vs. CD14^-^ PBMC), we identified genes already recognized for its predominant expression in CD14^+^ PBMC (CCL2, TLR2/4) or CD14^-^ PBMC (CCL5, PRF1) (18) but also genes that were not previously recognized as markers of circulating CD14^+^ PBMC, namely two members of EGR family of transcription factors (EGR1, EGR2) and CD36, a transporter of fatty acids. These findings are generally in accordance with the recently published dataset by Monaco et al. who performed RNASeq in 29 subpopulations of PBMC (19).

Although correction for multiple testing of mRNA levels in the basal state revealed no differences between experimental groups in either PBMC fraction, according to individual Mann Whitney comparison, we found rather contra intuitive alterations of IRS1, TLR2 and TNFα expression in CD14^+^ PBMC from FDR when compared with cells from controls. Higher mRNA levels of IRS-1, which we found in CD14^+^ PBMC from FDR, are linked to higher insulin sensitivity and fewer vascular complications in adipose tissue (20), i.e. to better metabolic and cardiovascular health. Nevertheless overall levels of IRS1 in monocytes were very low and together with undetectable levels of GLUT4, it is unlikely that this difference in IRS1 expression in CD14^+^ PBMC from FDR could have a major impact on glucose metabolism in FDR. Similarly, lower expression of pro-inflammatory TNFα and TLR2 detected in monocytes from FDR could be considered as a sign of lower inflammation but lower expression of these genes was not associated with increased insulin resistance as evaluated by HOMA-IR and Matsuda index (not shown).

FDR CD14^-^ PBMC exhibited also lower expression of ATGL, a lipase that was found to regulate phagocytosis by macrophages (21) and the production of arachidonic acid in neutrophils and mast cells (22). Although the role of ATGL in CD14^-^ PBMC remains unknown, ATGL mRNA levels in CD14^-^ PBMC correlated strongly with mRNA for genes involved in lipogenesis (FASN, ACACA) as well as genes implicated with β oxidation (ACOX1, **Figure 1C**). This nicely supports the existence of a newly discovered metabolic interplay among lipogenesis, β oxidation, and lipolysis in lymphocytes (abundant in CD14^-^ fraction of PBMC), which appears to be necessary for their activation, proliferation, and differentiation, as fatty acids serve not only as a source of energy but are also important building blocks for membrane synthesis required for lymphocyte function and survival (23).

Results of our study suggest that FDR could differ from controls by the response of gene expression in circulating CD14^+^ and CD14^-^ PBMC to OGTT. Although the alteration of the dynamic response to glucose challenge in FDR was more pronounced in CD14^-^ PBMC, two genes expressed predominantly in CD14^+^ PBMC, i.e. CCL2 and EGR2, were dysregulated in FDR vs. controls in response to OGTT. EGR2 belongs to a zing-finger containing family of transcription factors and plays a role in the activation of monocytes and polarization of macrophages (24, 25). CCL2 is a well–known chemoattractant for CD14^+^ PBMC, triggering their infiltration to tissues and differentiation into macrophages (26). Thus, the dysregulation of CCL2 expression in FDR could contribute to the increased infiltration of CD206 negative monocytes/macrophages into adipose tissue, which we observed previously in response to experimental hyperglycaemia in obese women (27). In fact, regulation of both, CCL2 and EGR2 mRNA expression, by higher concentrations of glucose was previously demonstrated on the model of THP1 macrophages (28) and on PBMC in newly diagnosed pediatric patients with T1DM and T2DM suffering from hyperglycaemia (3). Thus, the higher expression of EGR2 in response to OGTT in FDR could lead to an aberrant immune activation of monocytes after meals rich in simple carbohydrates.

In CD14^-^ PBMC, glucose challenge uncovered an immunity-related gene PTGS2 with potentially differential behavior in FDR compared to controls. PTGS2 is involved in the conversion of arachidonic acid to prostaglandins regulating the immune response to inflammation (29). In T lymphocytes, PTGS2 expression is induced upon T cell receptor activation by nuclear factor of activated T-cells (NFAT) C2 (30, 31). Since NFAT transcription factors were shown to be glucose responsive in various cells, including β cells producing insulin (32), PTGS2 upregulation could be dependent on NFAT glucose sensing. Interestingly, the changes induced by OGTT in PGST2 were correlated with those of IL8, a well-known pro-inflammatory cytokine supporting the recruitment and infiltration of neutrophils and T cells into local inflammatory sites (33) and with glucose levels in 2 h OGTT (**Figure 1C**). This suggests that not only CD14^+^ but also CD14^-^ PBMC of FDR could be predisposed to pro-inflammatory activation by glucose challenge.

Nevertheless, majority of genes with differential behavior in CD14^-^ PBMC in response to glucose challenge in FDR vs. control were associated with lipid metabolism. Despite the fact that the regulation of lipid metabolism appears to be crucial for the activation of lymphocytes (34), not much is known about the possible link between glucose challenge, T2DM development and lymphocyte activation. First, we observed an alteration of OGTT-induced response of DGAT2, a gene involved in lipogenesis, in FDR. Lipogenesis has been recently demonstrated to be required for the survival, proliferation, and differentiation of various types of lymphocytes (23, 34). Expression of DGAT2, the enzyme catalyzing the terminal step in triglyceride synthesis, was shown to be higher in leucocytes from T2DM patients and to correlate with plasma glucose levels (35). Thus, it could be envisioned that the higher glucose and insulin levels seen in FDR (vs. controls) at the end of OGTT might stimulate lipogenesis in lymphocytes through activation of SREBPs. These transcription factors are, similarly to NFAT, expressed in lymphocytes and function as sensitive glucose sensors (36).

Next, we found that not only lipogenesis but also lipid efflux could be dysregulated in FDR lymphocytes since the expression of ABCA1, a transporter enabling cholesterol export from the cells to HDL particle (34), was lower in FDR lymphocytes in basal conditions, and it was less suppressed in response to glucose challenge. These results are in line with similar findings described previously in macrophages - the deficit of ABCA1 was detected in macrophage-derived foam cells and peritoneal macrophages of diabetic mice (37) and human macrophages in response to glucose (38). Although the data on the role of ABCA1 or cholesterol efflux in lymphocytes are scarce, the impairment of cholesterol efflux has been shown to stimulate the proliferation of CD4^+^ T lymphocytes, which are implicated in the development of atherosclerosis (39).

The last two genes with higher OGTT-induced response in CD14^-^ PBMC from FDR were ACOX1 and MLCYD, i.e. genes implicated in β oxidation of fatty acids. ACOX1 is the first enzyme of β-oxidation while MLCYD reduces levels of malonyl CoA, a potent allosteric inhibitor of CPT1 activity, and thus indirectly stimulates β oxidation. Both enzymes are present prevalently in peroxisomes, which are involved in the metabolism of long-chain and very-long-chain fatty acids (40). Interestingly, peroxisomal oxidation has been suggested as a compensatory mechanism to metabolize straight medium- and long-chain fatty acids in cases of mitochondrial fatty acid β-oxidation defects (41), which were observed in cells from T2DM patients (42). Although peroxisomes have been identified as pivotal regulators of immune function and inflammation (40), the detailed consequences of altered peroxisomal β oxidation in lymphocytes have not been thoroughly investigated yet. Together, as lipid synthesis and accumulation tend to drive a pro-inflammatory phenotype, while pathways augmenting β-oxidation and lipid efflux support an anti-inflammatory phenotype of immune cells (34), the assessment of lipid content and analysis of lipid turnover in FDR CD14^-^ PBMC in further studies is of great interest.

Although this is one of the rare studies enabling the assessment of the nutrient challenge in circulating CD14^+^ and CD14^-^ PBMC in human subjects with or without T2DM family background, it is, at the same time, subject to several limitations. Namely, it is a low number of subjects (given by the thorough matching of the two groups and insufficient RNA amount isolated from CD14^+^ PBMC). Indeed, correction for multiple testing revealed the lower statistical power of the results (higher risk of false discovery), which is probably due to the combination of low number of subjects and large number of analyzed genes. Another limitation is the fact that only one sex (men) was included in the study despite evidences that OGTT- induced effects may be sex-dependent (43). In addition, the splitting of PBMC into only two populations of cells limited the interpretations as possible variations in the gene expression of individual lymphocyte subtypes, dendritic and NK cells, which could not be detected by this study, might have important functional consequences on immunity but also the development of T2DM. Nevertheless, the separation of PBMC into two major subpopulations represents a simple strategy to obtain cells of innate vs. adaptive immunity. Finally, predisposition to T2DM was based only on the family history of T2DM and was not assessed by the analysis of risk variants of alleles known to contribute to T2DM development. Thus, we cannot exclude that these subjects could be predisposed by the exposition to the same environmental factors as their family members with T2DM. In every way, FDR included in the study exerted signs of worsened glucose metabolism showing that the increased risk of T2DM in these subjects is biologically relevant.

In conclusion, although limited by a lower number of examined subjects, our data suggest an altered basal and glucose-intake-induced response of expression of genes involved in the control of inflammation and lipid metabolism in both CD14^+^ (mostly monocytes) and CD14^-^ (mostly lymphocytes) PBMC in FDR of T2DM patients when compared with controls. Importantly, the gene regulation appeared to be PBMC-fraction dependent: it was different in CD14^+^ PBMC when compared with CD14^-^ PBMC. This suggests essential differences in metabolism of CD14^+^ PBMC when compared with CD14^-^ PBMC and points at pathways sensitive to the nutritional challenge represented by glucose intake. Future studies on cell-specific gene regulation of PBMC in larger groups of subjects are warranted.

## Data Availability Statement

The raw data supporting the conclusions of this article are presented in **Supplementary Table 2**.

## Ethics Statement

The studies involving human participants were reviewed and approved by Ethics committee of Third Faculty of Medicine, Charles University. The patients/participants provided their written informed consent to participate in this study.

## Author Contributions

MK performed isolation of PBMC, gene expression analysis, and contributed to writing of the manuscript. MŠi prepared the concept of the study and contributed to data analysis. VeŠ contributed to gene expression analysis. MŠt recruited volunteers and performed OGTT. EK recruited volunteers and performed OGTT. VlŠ prepared the concept of the study and contributed to the writing of the manuscript. LR contributed to data analysis, interpretation of results, and writing of manuscript. LR is a guarantor of this work and, as such, had full access to all the data in the study and takes responsibility for the integrity of the data and the accuracy of the data analysis. All authors contributed to the article and approved the submitted version.

## Funding

The work was supported by grant GACR 16-14048S of the Grant Agency of the Czech Republic, projects 260387/SVV/2018, and PROGRES Q36 of Charles University.

## Conflict of Interest

The authors declare that the research was conducted in the absence of any commercial or financial relationships that could be construed as a potential conflict of interest.

## Supplementary Material

Supplementary Table 1 List of analysed genes. For all analysed genes gene symbols, name of genes, TaqMan assay ID, median level and 75^th^–25^th^ percentile range of expression (2^−ΔCt^), and the ratio of expression between CD14^+^ and CD14^-^ PBMC are shown. Differences in expression between CD14^+^ and CD14^-^ PBMC (joint data of control and FDR subjects) were evaluated by paired Multiple Wilcoxon matched pairs signed rank tests, with multiple comparisons by False Discovery Rate (method by Benjamini, Krieger, and Yekutieli). ND, not detected. Level of significance for both p and q value was set to <0.05.

Supplementary Table 2 mRNA levels-raw data. The output from Fluidigm Biomark instrument is shown, i.e. the values represent Ct values. FDR, first degree relatives of T2DM patients; NTC, non template control.

Supplementary Figure 1 Evolution of glucose and insulin levels during OGTT. Data show means and SEM. Log2 transformed data were analysed by Two Way ANOVA with Sidak multiple comparison test. G-effect of group, T-effect of time, GxT-interaction, posthoc tests * p<0.05.
