# Supplementary figures and images for: Signs of Deregulated Gene Expression Are Present in Both CD14+ and CD14- PBMC From Non-Obese Men With Family History of T2DM
Source: Front Endocrinol (Lausanne). 2021 Feb 15;11:582732. doi: 10.3389/fendo.2020.582732 (PMC7917286; doi:10.3389/fendo.2020.582732)

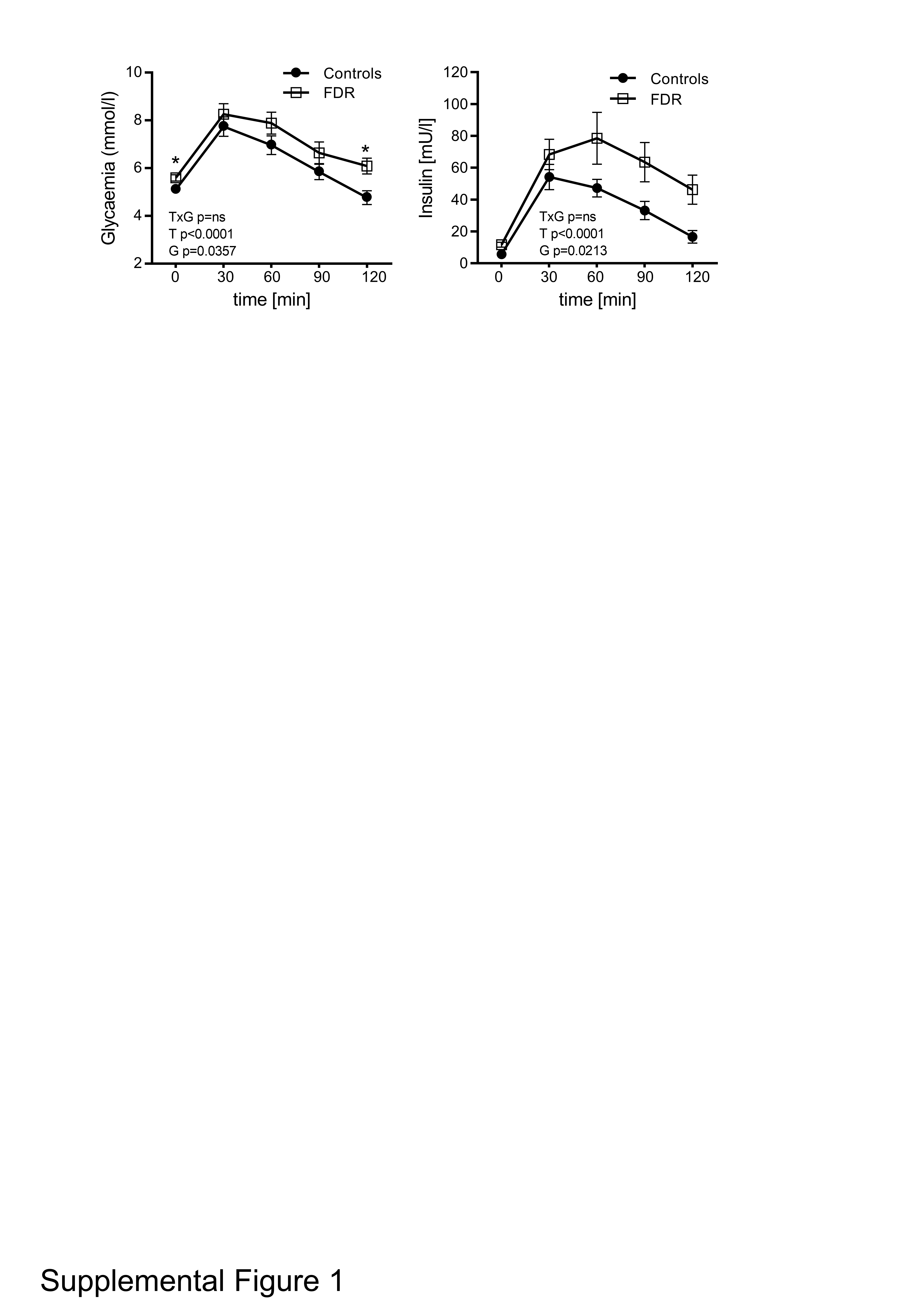

Supplement: Supplementary Figure 1 — Evolution of glucose and insulin levels during OGTT. Data show means and SEM. Log2 transformed data were analysed by Two Way ANOVA with Sidak multiple comparison test. G-effect of group, T-effect of time, GxT-interaction, posthoc tests * p<0.05. [file Image_1.tif]
